# A Mobile Anchor Assisted Localization Algorithm Based on Regular Hexagon in Wireless Sensor Networks

**DOI:** 10.1155/2014/219371

**Published:** 2014-07-13

**Authors:** Guangjie Han, Chenyu Zhang, Jaime Lloret, Lei Shu, Joel J. P. C. Rodrigues

**Affiliations:** ^1^Department of Information & Communication Systems, Hohai University, Changzhou 213022, China; ^2^Guangdong Provincial Key Lab of Petrochemical Equipment Fault Diagnosis, Guangdong University of Petrochemical Technology, Maoming 525000, China; ^3^Integrated Management Coastal Research Institute, Universidad Politecnica de Valencia, 46000 Valencia, Spain; ^4^Instituto de Telecomunicações, University of Beira Interior, 6201-001 Covilhã, Portugal

## Abstract

Localization is one of the key technologies in wireless sensor networks (WSNs), since it provides fundamental support for many location-aware protocols and applications. Constraints of cost and power consumption make it infeasible to equip each sensor node in the network with a global position system (GPS) unit, especially for large-scale WSNs. A promising method to localize unknown nodes is to use several mobile anchors which are equipped with GPS units moving among unknown nodes and periodically broadcasting their current locations to help nearby unknown nodes with localization. This paper proposes a mobile anchor assisted localization algorithm based on regular hexagon (MAALRH) in two-dimensional WSNs, which can cover the whole monitoring area with a boundary compensation method. Unknown nodes calculate their positions by using trilateration. We compare the MAALRH with HILBERT, CIRCLES, and S-CURVES algorithms in terms of localization ratio, localization accuracy, and path length. Simulations show that the MAALRH can achieve high localization ratio and localization accuracy when the communication range is not smaller than the trajectory resolution.

## 1. Introduction

Wireless sensor networks (WSNs) consist of a large number of sensor nodes deployed in a given region of interest (ROI) to fulfill tasks such as area surveillance, biological detection, home care, object tracking, and sending information to sink nodes via multihop communication [1–4].

In WSNs, determining unknown nodes' locations is a critical task since it provides fundamental support for many location-aware protocols and applications, such as location-based routing protocol; the location information is critical for sensor nodes to make optimal routing decisions [5, 6].

The problem of localization is a process of finding location information of the unknown nodes in a given coordinate system [7–9]. Based on the distance measurement technique used, localization algorithms can be classified into range-based localization algorithms and range-free localization algorithms. The range-based localization means that distances between sensor nodes are estimated by using physical properties of communication signal, that is, received signal strength indicator (RSSI), time of arrival (ToA), time difference of arrival (TDoA), and angle of arrival (AoA) [10]. Range-free localization algotihms do not need the distance or angle information for localization [11].

**Figure 1:**
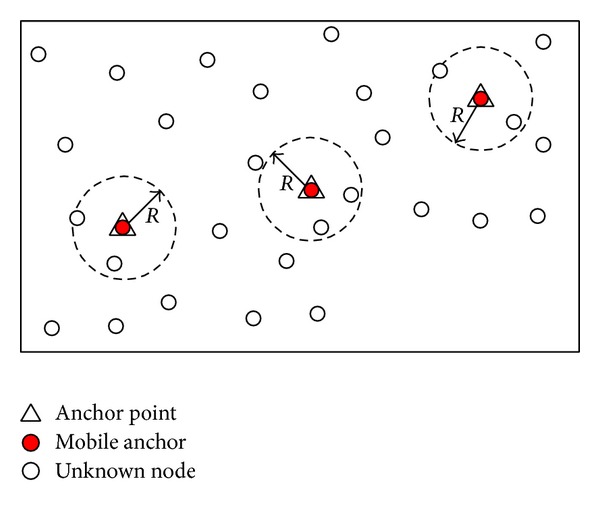
Mobile anchor assisted localization.

Constraints of cost and power consumption make it infeasible to equip each sensor node in the network with a GPS unit, especially for large-scale WSNs. A promising method to localize unknown nodes is to use several mobile anchors which are equipped with GPS units moving among unknown nodes and periodically broadcasting their current locations (beacon points) to help nearby unknown nodes with localization [12–15], as shown in Figure 1. This kind of architecture offers significant practical benefits, since the mobile anchor node is not as energy constrained as an unknown node and the localization accuracy also can be improved by carefully designing the mobile anchor's movement trajectory. Moreover, the size of a robot is much larger than the size of a sensor and thus it is much easier to install a GPS unit on it [16].

Generally, mobile anchor assisted localization algorithm involves three stages: (i) mobile anchor traverses the ROI while periodically broadcasting beacon packets which include their current positions; (ii) unknown nodes within the communication ranges of the mobile anchors receive the beacon packets and estimate distances to the anchors by using physical properties of communication signal when needed; and (iii) unknown nodes calculate their positions if they fall inside the overlapping communication ranges of at least three (four) noncollinear (noncoplanar) anchor nodes by using appropriate localization schemes in two-dimensional (2D) (three-dimensional (3D)) WSNs.

In this paper, we propose a mobile anchor assisted localization algorithm based on regular hexagon (MAALRH) with objectives of maximizing localization ratio and localization accuracy. To cover the entire ROI, we present a boundary compensation method (BCM) to ensure that the unknown nodes could fall inside the overlapping communication ranges of at least three noncollinear beacon points.

The rest of this paper is organized as follows.  Section 2 gives an overview of mobile anchor assisted localization algorithms.  Section 3 describes network model and theoretical background.  Section 4 introduces the proposed MAALRH and the comparing algorithms. Simulation results and performance analysis are shown in Section 5. Finally, Section 6 concludes this paper and discusses future research issues.

## 2. Related Work

### 2.1. Path Planning Scheme

A fundamental research issue of mobile anchor assisted localization algorithm is to design path planning scheme that mobile anchor should move along in a given ROI in order to minimize the localization error as well as the time required to localize the whole network.

Path planning schemes can be either static or dynamic. Static path planning scheme designs movement trajectory before starting execution; mobile anchor follows the predefined trajectory during the localization process. Dynamic path planning scheme designs movement trajectory dynamically or partially according to the observable environments or deployment situations and so forth.

#### 2.1.1. Static Path Planning Scheme

Koutsonikolas et al. [17] proposed SCAN, DOUBLE-SCAN, and HILBERT to satisfy network coverage. Movement trajectories of SCAN and DOUBLE-SCAN are composed of a series of straight lines. HILBERT curve divides the 2D area into square cells and connects the centers of those cells using line segments. Compared with SCAN and DOUBLE-SCAN, the HILBERT can provide more noncollinear beacon points for unknown nodes. To reduce the collinearity during localization, Huang and Záruba proposed two path planning schemes, namely, CIRCLES and S-CURVES. CIRCLES consists of a sequence of concentric circles centered within a ROI. S-CURVES is based on SCAN, which progressively scans the monitoring area from left to right taking an “*S*” curve. Hu et al. [19] proposed a mobile anchor centroid localization (MACL) method. The mobile anchor traverses the monitoring area following a spiral trajectory while periodically broadcasting beacon packets which contain its current position and so forth. Zhang et al. [20] proposed a collaborative localization scheme using a group of mobile anchor nodes (GMAN). A GMAN is composed of three anchor nodes, which form an equilateral triangle and each anchor node locates at one of the three vertexes. Cui et al. [21] introduced five movement trajectories for 3D WSNs. LAYERED-SCAN and LAYERED-CURVE divide the 3D ROI into several layers along one axis and regard each layer as a 2D ROI. Thus, in each layer of LAYERED-SCAN and LAYERED-CURVE, the mobile anchor traverses along one dimension using SCAN and S-CURVES, respectively. TRIPLE-SCAN and TRIPLE-CURVE divide the ROI into several layers along three axes. 3D HILBERT has more turns compared with LAYERED-SCAN and TRIPLE-SCAN to overcome collinearity and coplanarity problems. Cui and Wang [22] proposed a four-mobile-beacon assisted weighted centroid localization method. The four mobile beacons form a regular tetrahedron and traverse the given ROI following the LAYERED-SCAN trajectory which consists of several parallel layers of SCAN.

#### 2.1.2. Dynamic Path Planning Scheme

A large amount of dynamic path planning schemes were proposed to consider the real distribution of unknown nodes in the given ROI.

Li et al. [23] regard a WSN as a connected undirected graph. They proposed a Breadth-First (BRF) algorithm and a Backtracking Greedy (BTG) algorithm to transform the path planning issue into seeking spanning trees of the undirected graph and traversing through the graph. Thus, the movement trajectory of the mobile anchor node changes dynamically accordingly to the distribution of unknown nodes. Fu et al. proposed a novel dynamic movement trajectory based on virtual force, which is constructed by interaction force between mobile anchor and unknown nodes [24]. Each unknown node is equipped with an omnidirectional antenna. The mobile anchor uses directional antennas to receive feedback messages from unknown nodes and calculates the total virtual force on itself. In [25], three mobile anchors form a regular triangle with the length of its communication range and move in a ROI. Unknown nodes that do not know their own positions request the mobile anchor to deliver more beacon messages. The mobile anchor decides its movement trajectory on the basis of the received request messages. In [26], six optional positions are provided to be chosen based on geometry. The mobile anchor finds a new position among the six optional positions. The unknown node with most neighbors has the most chance to be the next position of the mobile beacon.

### 2.2. Localization Scheme

Another research issue of mobile anchor assisted localization algorithm is to design localization scheme by which unknown nodes calculate their positions based on beacon points received from mobile anchors.

Ssu et al. [27] developed a localization mechanism using the geometry conjecture, that is, perpendicular bisector of a chord. If any two chords are obtained, the location of the sensor node can be easily computed based on the conjecture. In [28], instead of using the absolute RSSI values, by contrasting the measured RSSI values from the mobile beacon to a sensor node, perpendicular intersection (PI) utilizes the geometric relation of PI to compute the position of the node. Guerrero et al. [29] intruded an azimuthally defined area localization (ADAL) algorithm which utilizes a beacon with a rotary directional antenna to send message in a determined azimuth periodically, and an unknown node uses the centroid of intersection area of several beacon messages as its position. Arrival and departure overlap (ADO) [30] uses a possible area delimited by two circles with the same radius at different centers. To estimate its position, an unknown node should obtain prearrival position, arrival position, departure position, and postdeparture position of the moving beacon to compute its ADO. To improve the localization accuracy of Ssu's scheme, Lee et al. [31] proposed a method based on geometric constraints utilizing three beacon points, where two are used for obtaining the intersection area and the third is used further to delimit this area. The borderline measurement schemes determine some straight lines that pass through a sensor node and use the intersection point of these lines as its position.

## 3. Network Model and Theoretical Background

### 3.1. Network Architecture and Assumptions

**Figure 2:**
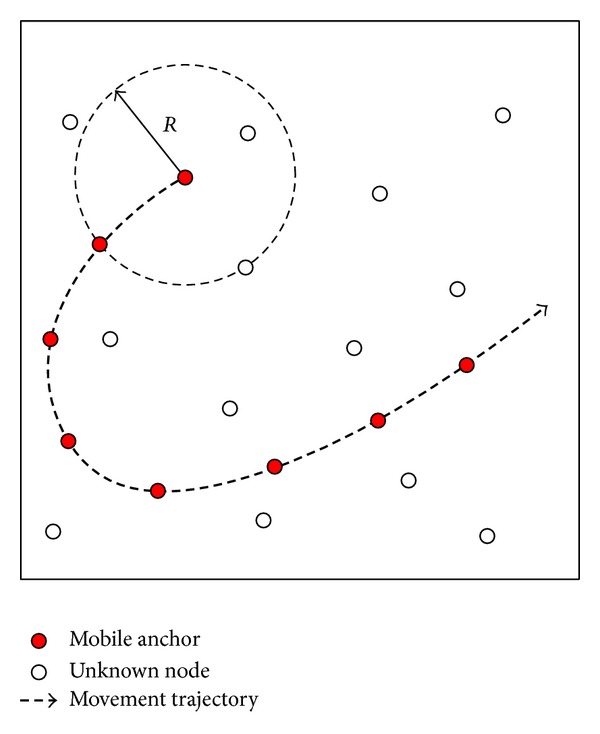
Network architecture.

Network architecture of this paper is shown in Figure 2. There are two types of sensor nodes in the network, namely, unknown node and mobile anchor. All the sensor nodes have the same communication rang. Unknown nodes are deployed uniformly in the ROI. The mobile anchor travels among unknown nodes following the predefined trajectory while periodically broadcasting its current location to help nearby unknown nodes with localization. Unknown nodes estimate distances to the mobile anchor by using RSSI technique. Once an unknown node receives at least three noncollinear anchor points, it will calculate its position by using trilateration method.

Two assumptions are made.

- The mobile anchor has sufficient energy for moving and broadcasting anchor packets during localization. The speed of the mobile anchor is adjustable and uniform in the process of localization.
- The communication model is perfect spherical radio propagation and there exists measurement errors. The mobile anchor has identical communication range *r* at all anchor points. Only the sensors within the communication range are assumed to be able to receive anchor packets sent by the mobile anchor.

### 3.2. Theoretical Background

In a two-dimensional ROI, suppose that the unknown node *p*(*x*_0_, *y*_0_) can receive three anchor coordinates *p*_*i*_(*x*_*i*_, *y*_*i*_), *i* = 1,2, 3. Distances between *p* and *p*_*i*_ are *r*_*i*_, *i* = 1,2, 3. Assume that the measurement error ranges from −*ε*_*i*_ to *ε*_*i*_, *ε*_*i*_ > 0. Thus, we can obtain

(1) Cpi =(x,y) ∣ (ri−εi)2≤(x+xi)2+(y+yi)2≤(ri+εi)2,i=1,2,3.

Unknown node calculates its coordinates by using of the trilateration. Thus, the localization error is defined as

(2) $$\begin{array}{c} \text{area}\left(C_{p_{i}}\right)=\left(x,y\right)\;|\;x\in \overset{3}{\underset{i=1}{⋂}}C_{p_{i}},\;y\in \overset{3}{\underset{i=1}{⋂}}C_{p_{i}}. \end{array}$$

**Figure 3:**
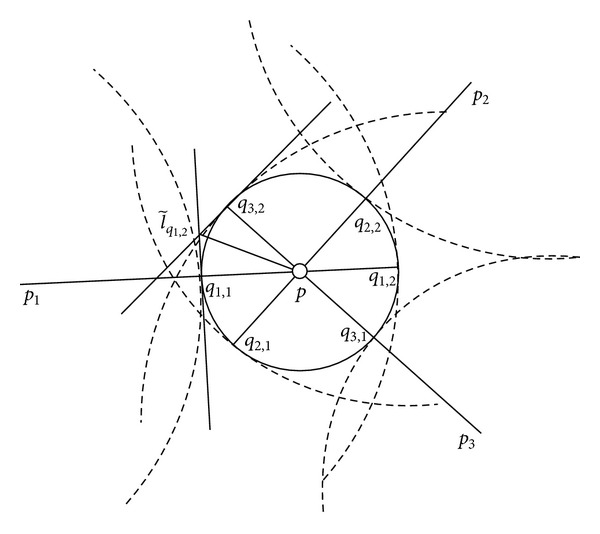
Analysis of localization error.

When the measurement error *ɛ* is relatively small, *C*_*p*_*i*__ can be linearized and approximated by ${\tilde{C}}_{p_{i}}$. We proved that the localization error is the smallest when three anchor nodes are placed symmetrically; namely, three anchor nodes form a regular triangle [32]. As shown in Figure 3, let *l*_*p*,*p*_*i*__ be the straight line passing through both *p* and *p*_*i*_. Thus, *l*_*p*,*p*_*i*__ will intersect with *S*_*p*_ at two points *q*_*i*,1_ and *q*_*i*,2_. For *j* = 1,2, we define the line passing through *q*_*i*,*j*_ and tangent to *S*_*p*_ as ${\tilde{l}}_{q_{i,j}}$. Therefore [32],

(3) $$\begin{array}{c} \text{area}\left(\tilde{C}\right)={2\varepsilon }^{2}\left(\tan\frac{\alpha_{1,2}}{2}+\tan\frac{\alpha_{2,3}}{2}+\tan\frac{\alpha_{3,1}}{2}\right). \end{array}$$

Note that

(4) $$\begin{array}{c} \alpha_{1,2}+\alpha_{2,3}+\alpha_{3,1}=\pi . \end{array}$$

Since (tan*x*)′′ = 2tan*x*(1 + tan*x*) ≥ 0, when 0 ≤ *x* ≤ *π*/2, we, thus, obtain

(5) area(C~)=6ε213(tanα1,22+tanα2,32+tanα3,12)≥6ε2tanα1,2+α2,3+α3,16=6ε2tanπ6.

The equality holds when

(6) $$\begin{array}{c} \alpha_{1,2}=\alpha_{2,3}=\alpha_{3,1}=\frac{\pi }{3}. \end{array}$$

In other words, the localization error is the smallest when three anchor nodes form a regular triangle.

## 4. Mobile Anchor Assisted Localization

The problem of path planning for mobile anchor is to design movement trajectory satisfying the following properties: (i) it should pass closely to as many potential node positions as possible, aiming at localizing as many unknown nodes as possible; (ii) it should provide each unknown node with at least three (four) noncollinear (noncoplanar) anchor points in a 2D (3D) WSN to achieve unique estimation of known node's position; (iii) it should be as short as possible to reduce the energy consumption of mobile anchors and time for localization.

The performances of mobile anchor assisted localization algorithm are influenced by the following factors.

- Communication range: a larger communication range of the mobile anchor covers more unknown nodes. Thus, the unknown nodes have more choices to select appropriate anchor points to calculate their coordinates.
- Movement trajectory: a well designed movement trajectory can eliminate collinearity (coplanarity) problem and make full use of the real-time information, that is, the distribution of unknown nodes, environment information, and so forth.
- Broadcast interval: a shorter broadcast interval means that the mobile anchor would broadcast its location more frequently, which may bring about a better localization performance.
- Path length: a longer path length means that the mobile anchor has more opportunities to broadcast its location and pass by more unknown nodes; however, it will consume more energy.

Thus, we should solve the above four problems when designing a mobile anchor assisted localization algorithm.

### 4.1. MAALRH

The general procedure of MAALRH consists of four steps, as shown in Algorithm 1.

#### 4.1.1. Network Segmentation

**Figure 4:**
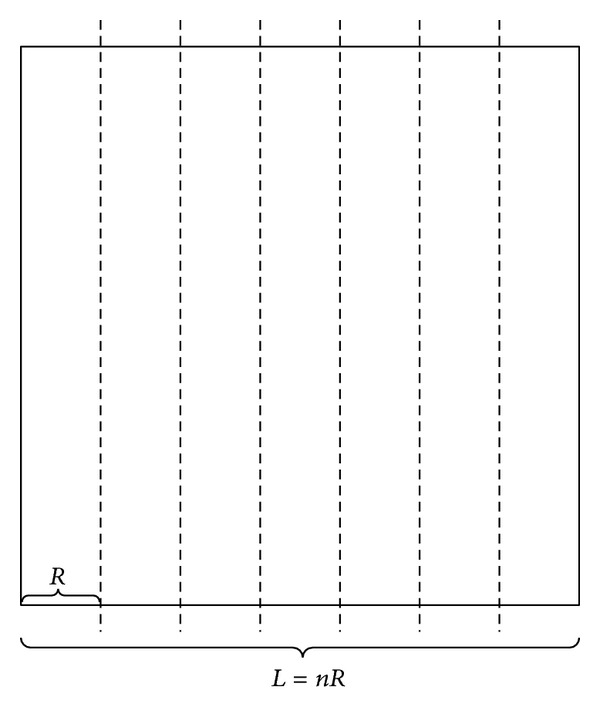
An example of network segmentation.

We assume that the ROI is a square. We divide the ROI into several subrectangles according to the length of the square. The communication range of mobile anchor nodes can be adjusted according to the length of subrectangles. The distance between two successive segments of the subrectangles is defined as the resolution (*R*).  Figure 4 gives an example of network segmentation. The length of the ROI is *L*. The square can be divided into *n* subrectangles which satisfy *L* = *nR*, *n* ∈ N*.

#### 4.1.2. Movement Trajectory

Assume that the ROI is a square with the area of *L* × *L*; the vertex coordinates of the ROI are (*x*_min⁡_, *y*_min⁡_), (*x*_max⁡_, *y*_min⁡_), (*x*_min⁡_, *y*_max⁡_), and (*x*_max⁡_, *y*_max⁡_), respectively. The mobile anchor is initially located at the centroid of the ROI. The initial coordinates of the mobile anchor can be calculated by using

(7) $$\begin{array}{c} x_{0}=\frac{\left|x_{\max}\right|-\left|x_{\min}\right|}{2}\\ y_{0}=\frac{\left|y_{\max}\right|-\left|y_{\min}\right|}{2}. \end{array}$$

**Figure 5:**
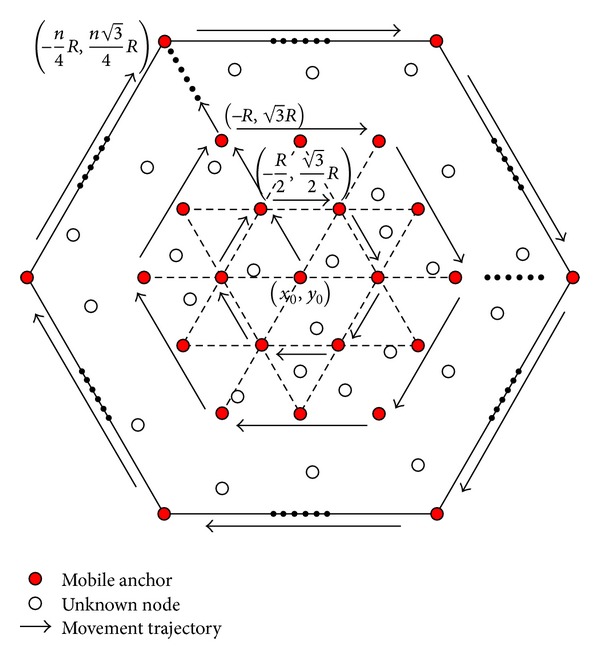
Movement trajectory of MAALRH without a boundary compensation method.

The mobile anchor traverses the entire ROI following the regular hexagon trajectory at the speed of *v* and broadcasts its current location (*x*_*i*_, *y*_*i*_) with an interval *T* and a communication range *r* as depicted in Figure 5.

A rectangular coordinate system is constructed with the origin at (*x*_0_, *y*_0_). At first, the mobile anchor moves from (*x*_0_, *y*_0_) to one of the vertexes of the regular hexagon with the side length of *R*; for instance, the mobile anchor moves from (*x*_0_, *y*_0_) to $\left(-\left(1/2\right)R,(\sqrt{3}/2)R\right)$. Then, the mobile anchor moves along the sides of the first regular hexagon with the side length of *R*. When the mobile anchor arrives at the point $\left(-\left(1/2\right)R,(\sqrt{3}/2)R\right)$ once again, it moves to $\left(-R,\sqrt{3}R\right)$ and then moves along the sides of the second regular hexagon with the side length of 2*R*. The side length of regular hexagons increases by *R* each time, and the mobile anchor traverses the ROI along the sides of *n*/2 regular hexagons. The cycle repeats until the mobile anchor arrives at the point $\left(-(n/4)R,(n\sqrt{3}/4)R\right)$ twice. Thus, the total path length without a boundary compensation method can be calculated by using

(8) $$\begin{array}{c} L_{\text{MAALRH}}^{\prime }=\frac{3}{4}n^{2}R+\frac{3}{2}nR=\frac{3}{4}\frac{L^{2}}{R}+\frac{3}{2}L. \end{array}$$

Thus, for a given ROI, the total path length depends on the *R*. A smaller *R* results in a lager path length. Since *R* = *vT*, with the same movement speed, the smaller *R* is, the less anchor packets are broadcasted. By this means, the broadcasted anchor points form many regular triangles.

#### 4.1.3. Boundary Compensation Method

**Figure 6:**
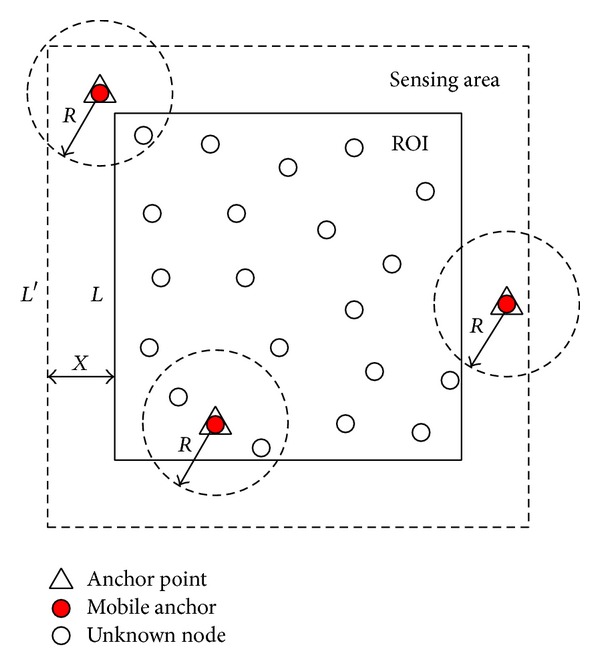
The relation of sensing area and ROI.

Since the regular hexagon movement trajectory leaves four corners of ROI uncovered, to improve localization ratio, we present a boundary compensation method to enhance the MAALRH. In BCM, mobile anchor travels in the sensing area which is larger than the ROI, as shown in Figure 6. Unknown nodes are deployed uniformly in the ROI while mobile anchor moves in the sensing area according to the movement trajectory. Assume that the length of the ROI is *L*, the length of the sensing area is *L*′, the relation of *L* and *L*′ can be expressed as *L*′ = *L* + *X*, *X* ∈ *R*_+_, and *X* is determined by the movement trajectory and the communication range of mobile anchor. In other words, by enlarging movement area of mobile anchor, the ROI can be traversed entirely. Therefore, unknown nodes which are at the boundary of the ROI can be localized.

**Figure 7:**
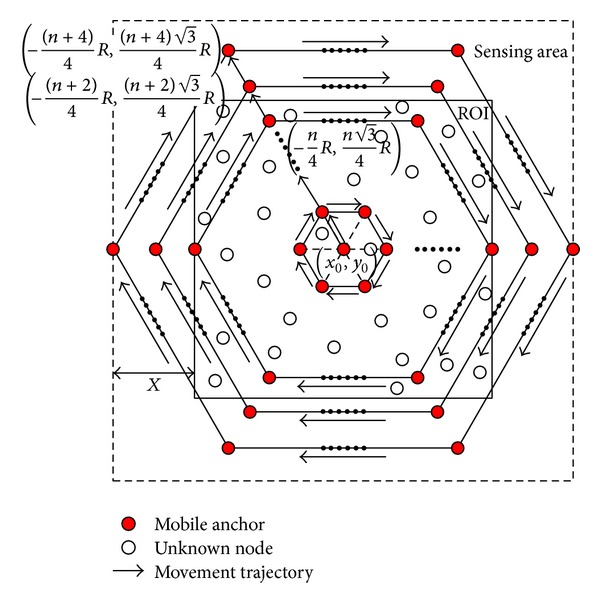
Movement trajectory of MAALRH with a boundary compensation method.

We choose *X* = 2*R* here. Thus, when the mobile anchor arrives at the point $\left((-n/4)R,(n\sqrt{3}/4)R\right)$ once again after it moves along the (*n*/2)th regular hexagon with the side length of 3*nR*, the mobile anchor moves to $\left(-((n+2)/4)R,((n+2)\sqrt{3}/4)R\right)$ and moves along the *n*/2 + 1th regular hexagon with the side length of (3*n* + 6)*R*. Then, the mobile anchor moves to $\left(-\left((n+4)/4\right)R,((n+4)\sqrt{3}/4)R\right)$ and moves along the *n*/2 + 2th regular hexagon with the side length of (3*n* + 12)*R* to ensure that unknown nodes at the boundary of the ROI could fall inside the overlapping communication ranges of at least three noncollinear anchors, as shown in Figure 7.

Thus, path length of the MAALRH with a boundary compensation method can be calculated by using

(9) $$\begin{array}{c} L_{\text{MAALRH}}=\frac{3}{4}n^{2}R+\frac{15}{2}nR+18R. \end{array}$$

#### 4.1.4. Trilateration

**Figure 8:**
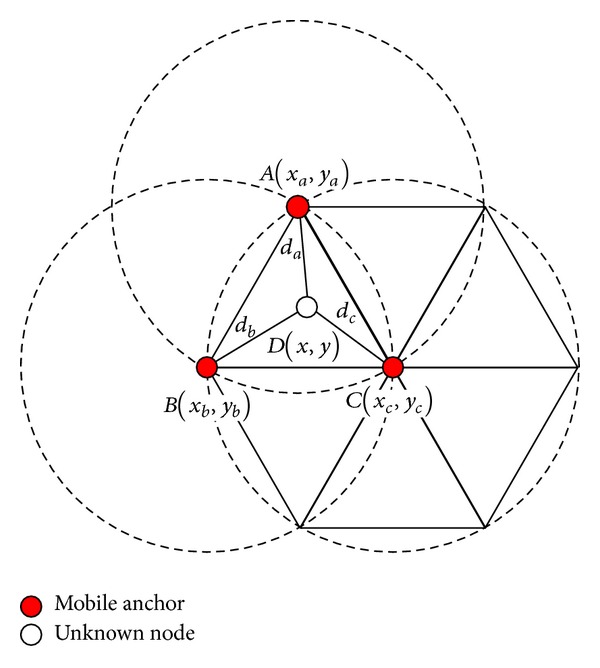
An example of the trilateration.

An example of the trilateration is shown in Figure 8. Suppose that the unknown node *D*(*x*, *y*) receives three anchor packets from the mobile anchor, namely, *A*(*x*_*a*_, *y*_*a*_), *B*(*x*_*b*_, *y*_*b*_), and *C*(*x*_*c*_, *y*_*c*_). Distances between *A*, *B*, *C*, and *D* are *d*_*a*_, *d*_*b*_, and *d*_*c*_, respectively. Since the unknown node *D* is within the regular triangle which is composed of *A*, *B*, and *C*, unknown node *D* will calculate its location by using

(10) $$\begin{array}{c} {\left(x-x_{a}\right)}^{2}+{\left(y-y_{a}\right)}^{2}=d_{a}^{2},\\ {\left(x-x_{b}\right)}^{2}+{\left(y-y_{b}\right)}^{2}=d_{b}^{2},\\ {\left(x-x_{c}\right)}^{2}+{\left(y-y_{c}\right)}^{2}=d_{c}^{2}. \end{array}$$

Hence,

(11) $$\begin{array}{c} \begin{array}{c} D{\left(x,y\right)}^{T}=\frac{1}{2}A^{-1}B, \end{array} \end{array}$$

where

(12) $$\begin{array}{c} A=\left(\begin{array}{cc} x_{a}-x_{c} & y_{a}-y_{c}\\ x_{b}-x_{c} & y_{b}-y_{c} \end{array}\right),\\ B=\left(\begin{array}{c} x_{a}^{2}-x_{c}^{2}+y_{a}^{2}-y_{c}^{2}+d_{c}^{2}-d_{a}^{2}\\ x_{b}^{2}-x_{c}^{2}+y_{b}^{2}-y_{c}^{2}+d_{c}^{2}-d_{b}^{2} \end{array}\right). \end{array}$$

### 4.2. Comparing Algorithms

Various path planning schemes have been proposed for single mobile anchor assisted localization. We choose HILBERT, CIRCLES, and S-CURVES to be compared with our proposed MAALRH.

#### 4.2.1. HILBERT

HILBERT can reduce the collinearity without significantly increasing the path length compared with SCAN and DOUBLE-SCAN. A* level*-*n* HILBERT curve divides the *L* × *L* ROI into 4^*n*^ square cells and connects the centers of those cells using 4^*n*^ line segments [17]. The resolution of the HILBERT curve is defined as the length of each line segment. Thus, *L*, *R*, and *n* satisfy 4^*n*^ = (*L*/*R*)×(*L*/*R*). Therefore, the path length of HILBERT curve can be calculated by using

(13) $$\begin{array}{c} L_{\text{HILBERT}}=n^{2}\times R. \end{array}$$

#### 4.2.2. CIRCLES

CIRCLES consists of a sequence of concentric circles centered within the ROI [18]. The resolution is defined as the diameter of the innermost circle. For each outer circle the radius is increased by *R* sequentially. CIRCLES can reduce collinearity of the anchor points; all areas within the concentric circles can be localized. However, CIRCLES leaves four corners of the ROI uncovered. The path length of CIRCLES can be calculated by using

(14) $$\begin{array}{c} L_{\text{CIRCLES}}=\frac{n^{2}\pi R}{4}+\left(\frac{n}{2}-1\right)R. \end{array}$$

#### 4.2.3. S-CURVES

S-CURVES is based on the SCAN, which progressively scans the ROI from left to right. However, S-CURVES takes an “*S*” curve instead of moving in a straight line [18]. For a *L* × *L* ROI and a resolution of *R*, there are ⌊2(*n* − 1)/3⌋ + 1 curve. Each vertical “*S*” curve consists of *n* − 1 semicircle with the radius of *R*/2. Therefore, the path length of S-CURVES can be calculated by using

(15) LS-CURVES=(n−1)πR2(⌊2(n−1)3⌋+1) +(n−2)R+πR2.

## 5. Performance Evaluation

### 5.1. Evaluation Criteria

*Localization Ratio*. Localization ratio is the ratio of the number of localizable unknown nodes to the number of unknown nodes. This metric also indicates the coverage degree of the movement trajectory. Localization ratio is defined as

(16) $$\begin{array}{c} L_{\text{ratio}}=\frac{N_{l}}{N_{o}}, \end{array}$$

where *N*_*l*_ is the number of localizable unknown nodes and *N*_*o*_ is the number of unknown nodes.

*Localization Accuracy.* The localization error of unknown node *i* is defined as

(17) $$\begin{array}{c} e_{i}=\frac{\sqrt{{\left(u_{i}-x_{i}\right)}^{2}+{\left(v_{i}-y_{i}\right)}^{2}+{\left(w_{i}-z_{i}\right)}^{2}}}{r}, \end{array}$$

where (*u*_*i*_, *v*_*i*_, *w*_*i*_) are real coordinates of an unknown node *i*, (*x*_*i*_, *y*_*i*_, *z*_*i*_) are estimated coordinates of an unknown node *i*, and *r* is the communication range of sensor nodes.

We evaluate the localization accuracy by using average and standard deviation of localization errors of unknown nodes, which are defined as

(18) $$\begin{array}{c} \mu_{e}=\frac{1}{N_{l}}\overset{N_{l}}{\underset{i=1}{\sum }}e_{i},\\ \sigma_{e}=\sqrt{\frac{1}{N_{l}}\overset{N_{l}}{\underset{i=1}{\sum }}{\left(e_{i}-\mu_{e}\right)}^{2}}, \end{array}$$

where *N*_*l*_ is the number of localizable unknown nodes in a WSN.

*Path Length.* To save energy consumption and time for localization, the path length of the mobile anchor node should be as short as possible.

*Scalability.* Scalability means that the localization performance is independent of the unknown nodes density.

### 5.2. Experiment Parameters

Our simulations are performed using Matlab. Suppose that the ROI is a square.  Table 1 lists parameters used in simulations. Five movement trajectories are compared in this section, HILBERT, CIRCLES, S-CURVES, MAALRH, and MAALRH_BCM (we name the MAALRH with the boundary compensation method as MAALRH_BCM). The trilateration is used to calculate coordinates of unknown nodes. To ensure reliability of evaluation results, 50 simulation runs were performed for each set of simulation condition, with a different uniform deployment of unknown nodes on each occasion.

### 5.3. Simulations and Analysis

We evaluate performances of five movement trajectories under three resolutions: 60 m, 80 m, and 120 m in terms of localization ratio, localization accuracy, path length, and scalability.

#### 5.3.1. Localization Ratio

**Figure 9:**
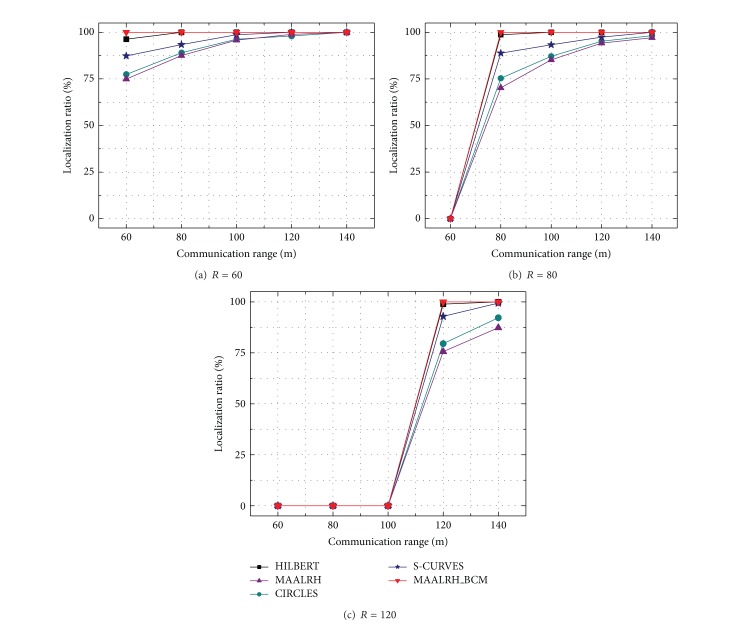
Localization ratio with different resolutions.

Figure 9 depicts the relation between localization ratios and communication ranges under three resolutions. The MAALRH_BCM outperforms HILBERT, CIRCLES, and S-CURVES and MAALRH in general. Localization ratios of MAALRH_BCM and HILBERT are similar when the communication range is not smaller than the resolution. Localization ratio of S-CURVES is larger than that of MAALRH and CIRCLES, since MAALRH and CIRCLES leave four corners of ROI uncovered. Localization ratio of MAALRH_BCM can reach 100% as long as the communication range is not smaller than the resolution, since unknown nodes can receive three noncollinear anchor points which can form a regular triangle to estimate their positions. When the resolution is relatively small, that is, *R* = 60 m, localization ratios increase rapidly with the increase of the communication range and the localization ratios of the five algorithms can all reach 100% when the communication range is 140 m. However, with the increase of the resolution, the mobile anchor dose not broadcast anchor packets as frequently as in the previous case (i.e., *R* = 60); only MAALRH_BCM and HILBERT can reach 100% localization ratio (i.e., *R* = 80 m and *R* = 120 m). That is because with the boundary compensation method MAALRH_BCM can provide noncollinear anchor points to ensure that the movement trajectory can cover the entire ROI. When the resolution is much larger than the communication range, unknown nodes cannot receive enough anchor points to estimate their coordinates, which results in zero localization ratios. For MAALRH and CIRCLES, the movement trajectories leave four corners of the ROI uncovered and unknown nodes on the boundary of the ROI cannot receive at least three noncollinear anchor packets from the mobile anchor, especially when the resolution is large. Thus, MAALRH and CIRCLES perform worse compared with the other three algorithms.

From the simulation, we can conclude that the localization ratio depends on the communication range of the sensor nodes and the resolution of the movement trajectory which determines the amount and the distance interval of the anchor points.

#### 5.3.2. Localization Accuracy

**Figure 10:**
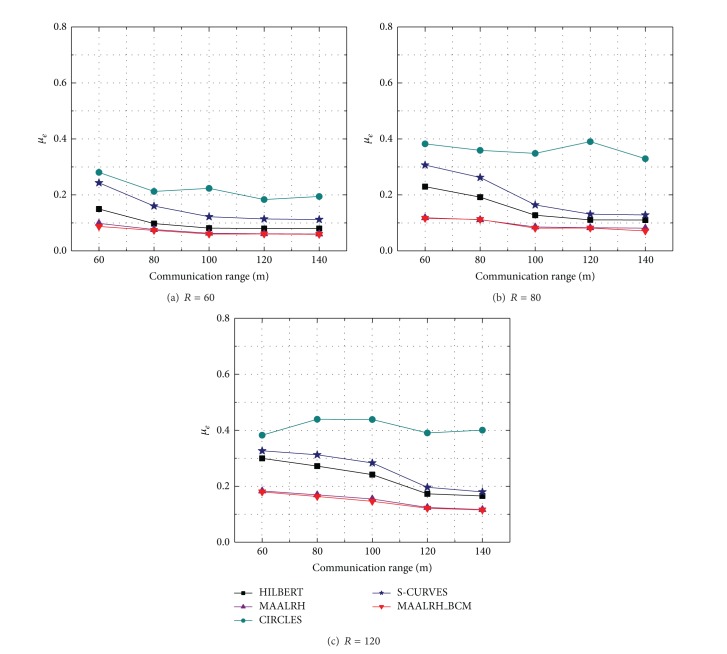
Average deviation with different resolutions.

Figure 10 presents the variation of *μ*_*e*_ under three resolutions. We can observe that with the increase of the resolution, *μ*_*e*_ increase correspondingly, because distance between two neighboring anchor points increases with the increase of the resolution and results in a larger measurement error. MAALRH and MAALRH_BCM almost have the same average deviation, since both of them use three anchor points which form a regular triangle to estimate unknown nodes' coordinates. Besides, the MAALRH_BCM has a boundary comprehension method to ensure the localization accuracy of all the unknown nodes within a ROI. HILBERT and S-CURVES have larger average deviations compared with that of MAALRH and MAALRH_BCM. CIRCLES performs worst among the five movement trajectories. Because in HILBERT, CIRCLES, and S-CURVES, ordinary nodes randomly select three noncollinear anchor points to calculate their positions. *μ*_*e*_ of MAALRH, MAALRH_BCM, HILBERT, and S-CURVES decrease apparently when the communication range is equal to the resolution (with the variation from 60 m to 140 m), because more unknown nodes can receive three noncoplanar anchor points. However, the movement trajectory of the CIRCLES leaves four corners of the ROI uncovered, which results in larger average deviations compared with other four algorithms.

**Figure 11:**
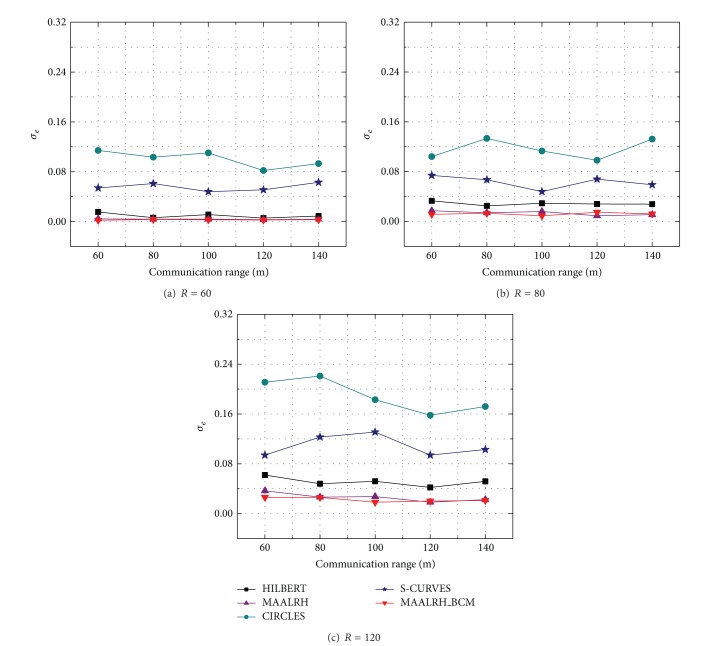
Standard deviation with different resolutions.

Figure 11 shows the standard deviation of the five mobile anchor assisted localization algorithms. MAALRH and MAALRH_BCM have the smallest *σ*_*e*_ compared with HILBERT, S-CURVES, and CIRCLES. From the simulation, we can draw the conclusion that the localization errors of MAALRH and MAALRH_BCM concentrate nearby the *μ*_*e*_, and the distribution of localization errors of HILBERT, S-CURVES, and CIRCLES is more dispersed than that of MAALRH and MAALRH_BCM, especially for CIRCLES.

#### 5.3.3. Path Length

**Figure 12:**
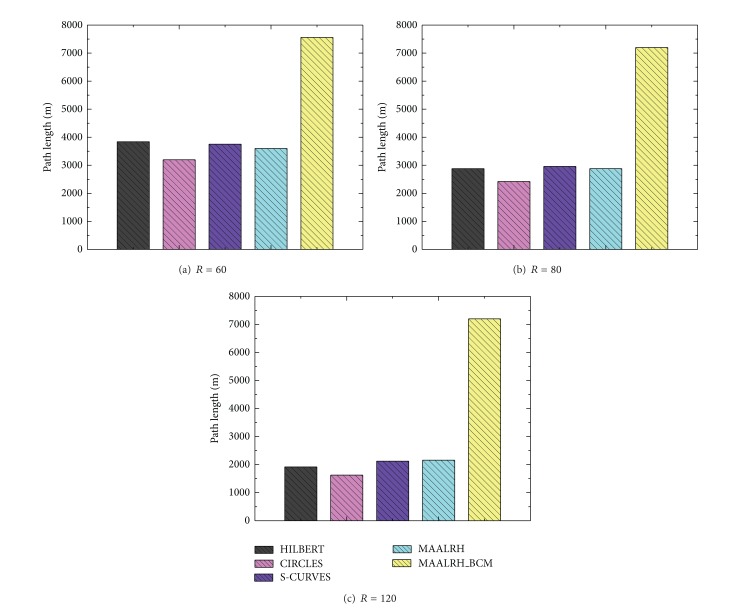
Path length with different resolutions.

For each of the five movement trajectories, the path length is a function of *n* and *R*. From Figure 12 we can observe that the CIRCLES has the shortest path length and MAALRH, HILBERT, and S-CURVES have the similar path lengths. The MAALRH_BCM has the longest path length, because the mobile anchor moves in the sensing area which is (*n* + 2)^2^ *R* ^2^ − *n* ^2^ *R* ^2^ larger than the ROI to ensure that unknown nodes which are deployed at the boundary of the ROI could fall inside the overlapping communication ranges of at least three noncollinear anchors. Thus, the path length of the MAALRH_BCM is 6*nR* + 18*R* longer than that of the MAALRH. The MAALRH_BCM sacrifices path length to maximize the localization ratio and the localization accuracy.

#### 5.3.4. Scalability

We vary the number of the unknown nodes from 100 to 500 with the step of 100, communication range of 80 m, and resolution of 80 m to test the scalability of the MAALRH_BCM.  Table 2 shows the relation between the localization ratio, *μ*_*e*_, *σ*_*e*_, and the number of unknown nodes. As depicted in Table 2, the localization ratio remains 100% with the increase of the number of unknown nodes; since the communication range is equal to the resolution, unknown nodes can receive three noncollinear anchor points which can form a regular triangle to estimate their positions. *μ*_*e*_ and *σ*_*e*_ change litter under different unknown node densities, where the maximum *μ*_*e*_ is 0.0214 m larger than the minimum *μ*_*e*_ and the maximum *σ*_*e*_ is 0.0071 m larger than the minimum *σ*_*e*_. This result proves that the localization ratio and localization accuracy of MAALRH_BCM do not depend on the unknown node density; they depend on the sensor node's communication range and mobile anchor's trajectory resolution which determine the amount and the distance interval of anchor points.

## 6. Conclusion

In this paper, we propose a mobile anchor assisted localization algorithm based on regular hexagon in two-dimensional WSNs, which can cover a square ROI entirely with a boundary compensation method. Simulations indicate that compared with HILBERT, CIRCLES, and S-CURVES algorithms, the MAALRH_BCM can achieve higher localization ratio and localization accuracy when the communication range is not smaller than the resolution. In summary, a carefully designed movement trajectory can significantly improve localization performances.

The future research issues in the area of mobile anchor assisted localization possibly are as follows.

- In real applications, obstacle-resistant mobile anchor assisted localization algorithms are needed to deal with the obstacles in a given ROI. Movement trajectories of mobile anchors should be designed dynamically or partially according to the observable environment or deployment situations to make full use of the real-time information during localization.
- Single mobile anchor assisted localization algorithm takes a long time to locate all the unknown nodes in a ROI, especially for a large-scale WSN. Thus, collaborative mobile anchor assisted localization algorithm which uses several mobile anchors should be specifically designed to reduce localization time and improve localization accuracy.

## Figures and Tables

**Algorithm 1:**
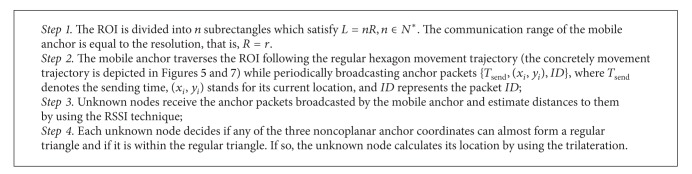
MAALRH algorithm.

**Table 1:** Parameters used in the simulation.

ROI size	480 m × 480 m
Communication range	60–140 m
Resolution	60 m, 80 m, and 120 m
Movement speed	10 m/s
Number of unknown nodes	100–500

**Table 2:** Scalability of MAALRH_BCM.

	100	200	300	400	500
*L*_ratio_	100%	100%	100%	100%	100%
μ_*e*_	0.1324	0.1538	0.1454	0.1459	0.1397
σ_*e*_	0.0237	0.0264	0.0193	0.0240	0.0204
